# Identification of 2′,4′-Dihydroxychalcone as an Antivirulence Agent Targeting HlyU, a Master Virulence Regulator in *Vibrio vulnificus*

**DOI:** 10.3390/molecules23061492

**Published:** 2018-06-20

**Authors:** Saba Imdad, Nayab Batool, Subhra Pradhan, Akhilesh Kumar Chaurasia, Kyeong Kyu Kim

**Affiliations:** Department of Molecular Cell Biology, School of Medicine, Samsung Medical Center, Sungkyunkwan University, Suwon 16419, Korea; elegantsb@yahoo.com; (S.I.); nayyab114@gmail.com (N.B.); subhrapradhan@gmail.com (S.P.)

**Keywords:** HlyU inhibitor, virulence factors, toxin-network, *Vibrio vulnificus*, 2′,4′-dihydroxychalcone, foodborne infectious disease, *Galleria mellonella* infection model, orthogonal screening reporter platform

## Abstract

The emergence of antimicrobial resistance and rapid acclimation allows *Vibrio vulnificus* to rapidly propagate in the host. This problematic pathological scenario can be circumvented by employing an antivirulence strategy, treating *Vibrio* infections without hindering the bacterial growth. We developed a genome-integrated orthogonal inhibitor screening platform in *E. coli* to identify antivirulence agents targeting a master virulence regulator of *V. vulnificus*. We identified 2′,4′-dihydroxychalcone (DHC) from the natural compound library and verified that it decreases the expression of the major toxin network which is equivalent to the ∆*hlyU* deletion mutant. 2′,4′-DHC also reduced the hemolytic activity of *V. vulnificus* which was tested as an example of virulence phenotype. The electrophoretic mobility shift assay confirmed that 2′,4′-DHC specifically targeted HlyU and inhibited its binding to P*_rtxA1_* promoter. Under in vivo conditions, a single dose of 2′,4′-DHC protected ~50% wax-worm larvae from *V. vulnificus* infection at a non-toxic concentration to both *V. vulnificus* and wax-worm larvae. In the current study, we demonstrated that an orthogonal reporter system is suitable for the identification of antivirulence compounds with accuracy, and identified 2′,4′-DHC as a potent antivirulence agent that specifically targets the HlyU virulence transcriptional regulator and significantly reduces the virulence and infection potential of *V. vulnificus*.

## 1. Introduction

*Vibrio vulnificus* is Gram-negative, halophilic, highly adaptable, opportunistic, and one of the most lethal human pathogens among foodborne infectious diseases [1]. *V. vulnificus* is predominantly present in seafood such as oysters, shrimps, fish, and shellfish [2]. Raw or undercooked seafood that is infected with *V. vulnificus* and then consumed by humans causes systemic infections such as lethal septicemia with an exceptionally high mortality rate surpassing 50% [3]. Owing to the extreme adaptability of this marine inhabitant, *V. vulnificus* can easily acclimatize to its human host and cause wound infections upon exposure to seawater, which may progress rapidly to cellulitis, ecchymosis, and finally result in necrotizing fasciitis [4,5]. *V. vulnificus* infections are one of the most life-threatening foodborne infectious diseases and are treated with quinolone, tetracycline, and cephalosporin antibiotics [6,7,8]. However, *V. vulnificus* strains have recently been reported as developing antimicrobial resistance (AMR) in many countries, against the antibiotics currently used for the treatment of *V. vulnificus* infections [9,10]. Therefore, it is necessary to develop new antibiotics or alternative therapeutic strategies for the treatment of AMR *V. vulnificus* infections. 

As an alternative approach to bactericidal antibiotics that cause AMR development, the antivirulence strategy is rapidly progressing to treat bacterial infections by disarming their virulence factors and subjecting them to be cleared by the host’s immune system [11,12]. The advantage of this approach is that such a strategy can be used not only for treating the AMR pathogens by targeting new therapeutic targets but also helps in reducing AMR development via antibiotic-mediated clonal selection. Therefore, it is necessary to develop antivirulence drugs targeting infection-induced virulence transcription factors/regulators for AMR *Vibrio* strains. 

The pathogenesis of *V. vulnificus* is tightly controlled by a master virulence transcriptional regulator/factor (VTF), HlyU, which is responsible for the induction of various virulence proteins including critical offensive toxins such as repeat-in-toxin (RtxA1) and hemolysin (VvhA) to establish successful infection via wound and intestinal routes [13,14,15,16]. In general, the master virulence regulator is an infection-induced transcription factor that regulates the genes responsible for switching the general physiology of microbes to pathophysiology and vice versa. In the present study, we aimed to target and inhibit the HlyU-based network of virulence factors including major cytolytic RtxA1 and VvhA. 

We developed an orthogonal genome-integrated, stable *Escherichia coli* inhibitor assay reporter platform for the identification of antivirulence compounds by screening the natural product library. The novel heterologous and isolated reporter platform identified 2′,4′-dihydroxychalcone (2′,4′-DHC) as an antivirulence agent that specifically targets HlyU and inhibits virulence factors under its control, similar to the *hlyU* deletion mutant (∆*hlyU*). In the present study, 2′,4′-DHC targeted HlyU and inhibited the virulence factors under its control to fight *V. vulnificus* pathogenesis under both in vitro and in vivo conditions using the *Galleria mellonella* (wax-worm) infection model.

## 2. Results

### 2.1. Development of Orthogonal Reporter Platform

**Figure 1:**
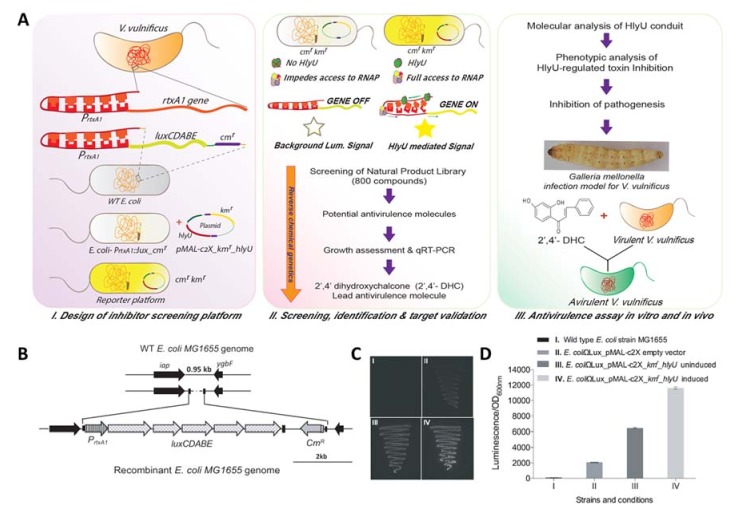
Development of a chromosomally integrated orthogonal inhibitor screening reporter platform and its evaluation, followed by the identification and validation of antivirulence inhibitor. (**A**) Overview of development of an orthogonal inhibitor/drug screening platform, screening, identification and validation of the antivirulence inhibitor; (**B**) schematic of integration for reporter cassette (P*_rtxA_*_1_*::luxCDABE-cm^r^*) in the intergenic region of *E. coli* using λ-red recombinase; (**C**) evaluation of reporter system by comparing luminescence signals. Various panels I–IV show: (I) wild-type *E. coli*; (II) reporter cassette, P*_rtxA_*_1_*::luxCDABE-cm^r^* integrated in the *E. coli* genome (*E. coli-*P*_rtxA_*_1_*::lux_cm^r^,* pMAL-c2X*_km^r^*), (III–IV) reporter platform (pMAL-c2X*_km^r^_hlyU* in *E. coli-*P*_rtxA_*_1_*::lux_cm^r^*) without (III), and with IPTG induction (IV) showing the HlyU dependent enhancement of luminescence signal; (**D**) quantitative analysis of luminescence/OD_600_ of the strains (I–IV).

The regulation of virulence genes by HlyU has been reported previously, wherein HlyU acts as an anti-repressor of the H-NS global repressor. As an example, H-NS binds to five H-NS binding sites of a major cytotoxin (RtxA1) promoter, P*_rtxA1_*, and keeps the *rtxA1* gene repressed under normal conditions [17] (Figure 1I). However, when *V. vulnificus* makes contact with the host cells, the transcription of *rtxA1* is induced several-fold upon host–pathogen interaction, as reported earlier [16,18]. HlyU binds to AT-rich regions ranging from −376 to −417 base pairs (bp) upstream of the transcription start site and de-represses the gene expression, allowing the translation of several virulence factors [17]. We utilized the known regulatory information to develop an orthogonal inhibitor screening platform to identify a small molecule inhibitor of the HlyU transcriptional regulator-based network of virulence factors of *V. vulnificus* (Figure 1 II & III). The advantages of an orthogonal reporter gene assay platform are as follows: (a) a non-native reporter gene is stably integrated at an innocuous site of the genome; and (b) there is no perturbation in the signal due to the cross-interaction of the gene-circuit installed in the reporter system.

To develop an orthogonal reporter system in K-12 MG1655 (hereafter referred to as *E. coli*), which was required for identifying the antivirulence compound against *V. vulnificus*, a 951 bp intergenic region of wild-type (WT) *E. coli* genome was chosen between two convergent genes, *iap* and *ygbF,* encoding an aminopeptidase and CRISPR (Clustered Regularly Interspaced Short Palindromic Repeats) adaptations RNA endonuclease, respectively. A DNA cassette P*_rtxA_*_1_*::luxCDABE_cm^r^* of 7997 bp from the pBBRMCS2_P*_rtxA_*_1_*::luxCDABE* plasmid (Figure S1A, Table S1) harboring the P*_rtxA_*_1_ promoter (754 bp) tagged with *luxCDABE* scorable reporter genes along with the chloramphenicol acetyltransferase (*cat*/*cm^r^*) gene was integrated by deleting a 0.4 kb region of the innocuous intergenic region of the *E. coli* genome, and the obtained recombinant clone was designated as *E. coli*-P*_rtxA_*_1_*::lux_cm^r^* (Figure 1B). The *hlyU* gene was expressed *in trans* under an isopropyl β-d-1-thiogalactopyranoside (IPTG) inducible P*_taclac_* promoter in the pMAL-c2X plasmid to mimic the higher level of expression of HlyU under pathophysiological condition. The ampicillin cassette was deactivated by inserting the kanamycin cassette at the ScaI site, and the resultant *hlyU* expression plasmid was designated as pMAL-c2X_*km^r^*_*hlyU* (Figure S1B, Tables S1 and S2). The *E. coli*-P*_rtxA_*_1_*::lux_cm^r^* strain with empty plasmid, pMAL-c2X_*km^r^*, showed minimal background luminescence compared to the WT *E. coli* (Figure 1C,D), whereas the pMAL-c2X_*km^r^*_*hlyU* in *E. coli-*P*_rtxA_*_1_*::lux_cm^r^* (reporter strain) showed leaky expression of HlyU that increased after IPTG induction (Figure 1C). The luminescence signal was found to be in proportion to the active HlyU level in the reporter strain measured qualitatively (Figure 1C) and quantitatively (Figure 1D). The instability of the multiple plasmid-based reporter platform is owing to the change in the copy number of plasmids and the inconsistent signal, which creates a problem and gives false positive results (Figure S2; for details, see Supporting Information). The reporter platform with a single integrated copy of scorable *lux* operon in the *E. coli*-P*_rtxA_*_1_*::lux_cm^r^* strain with *hlyU* expressing *in trans* yielded a stable and consistent signal in response to the presence of active HlyU. 

### 2.2. Screening, Identification, and Assessment of Potent Antivirulence Inhibitors from the Natural Product Library 

Eight-hundred compounds in the natural product library were screened for inhibitors of HlyU, a master VTF, using the newly developed orthogonal reporter platform (Figure 1 II). Considering the differential absorption, distribution, metabolism, and excretion (ADME) parameters for various compounds, a broad range of 10–40% luminescence inhibition per unit OD_600_ compared to their corresponding dimethyl sulfoxide (DMSO) carrier control was analyzed. Twenty-one hits were selected, of which 14 fell under the primary antivirulence hits category and seven belonged to known antimicrobial compounds (Table S3). Among the 14 antivirulence hits, resveratrol 4′-methyl ether and curcumin had already been reported as playing a role in inhibiting virulence through unknown targets, [19,20] and thus, the remaining 12 compounds were further investigated by evaluating the undesirable growth inhibition properties in the target organism *V. vulnificus* (Figure S3). Both purpurogallin-4-carboxylic acid and sanguinarine sulfate at 5 µM concentration showed significant growth inhibitory effects, which is evident by an extended lag-phase in the *V. vulnificus* growth-curve (Figure S3). Therefore, these two compounds were excluded in further analysis. 

**Figure 2:**
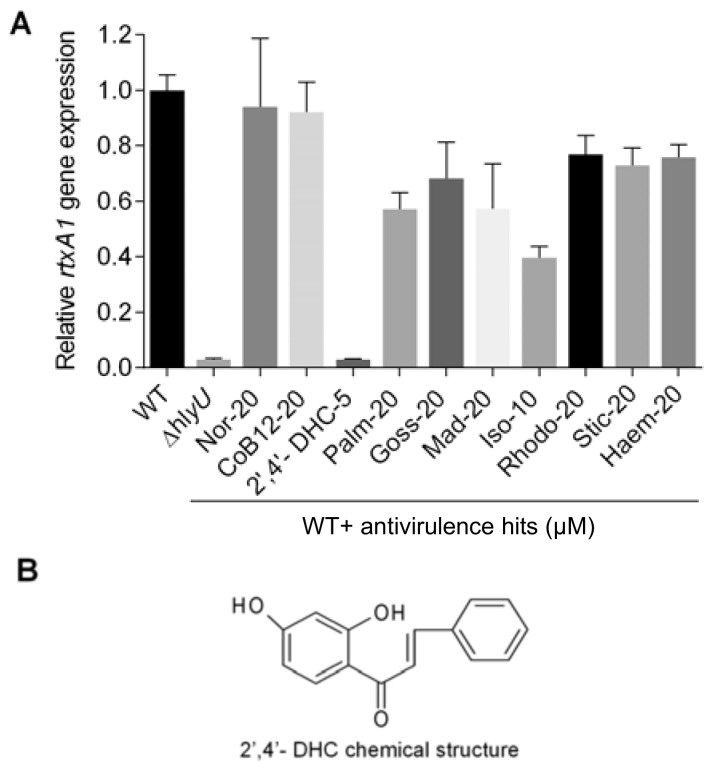
Identification and assessment of antivirulence 2′,4′- DHC. (**A**) *rtxA1* gene expression analysis of 10 secondary antivirulence hits. A fresh culture of wild-type (WT) *V. vulnificus* was incubated to grow with and without various compounds at the mentioned concentrations (µM) until OD_600_ 1.8–2.0 and total RNA was isolated to prepare cDNA for qRT-PCR analysis. Gene expression level was normalized with the expression level of gyrB. The relative expression level against the non-treated WT control sample is displayed; (**B**) chemical structure of 2′,4′-DHC*.*

Based on the growth response data of *V. vulnificus*, 2′,4′-DHC at 5 µM, isoliquiritigenin at 10 µM, and the remaining eight antivirulence hits at 20 µM were further analyzed to identify the potential antivirulence compounds using qRT-PCR of *rtxA1* gene (Figure 2A). Isoliquiritigenin, palmatine, and the remaining eight candidates showed modest 2.5-fold, 1.7-fold, and insignificant decrease in the rtxA1 transcript, respectively. In contrast, 2′,4′-DHC at 5 µM decreased the rtxA1 transcript by more than 30-fold compared to the DMSO carrier control (Figure 2A). Therefore, 2′,4′-DHC was selected for further studies as an antivirulence agent (Figure 2B). 

### 2.3. Toxicity of 2′,4′-DHC on the Mammalian Host Cell and V. vulnificus Growth 

Previously, 2′,4′-DHC has been reported to display antibacterial behavior towards various Gram-negative and Gram-positive bacteria. The reported MIC are much higher (>250–500 µg/mL for 2′,4′-DHC for various Gram-negative bacteria) [21] than the concentration tested in the present study. However, its effect on the growth of *V. vulnificus* has not been reported yet. We tested the MIC of 2′,4′-DHC against *V. vulnificus* and it was found to be between 64 and 128 µM (Figure S4B). The growth of *V. vulnificus* is not affected by 2′,4′-DHC up to 8 µM (Figure S4A). Therefore, 8 µM was set as the upper working concentration limit of 2′,4′-DHC for further experiments. 

The half maximal inhibitory concentration (IC50) of 2′,4′-DHC against HeLa and HEK293 cells was calculated to be 100.3 µM and 60.0 µM, respectively (Figure S5). The IC50 of 2′,4′-DHC against two mammalian cell lines was approximately three- to six-fold higher than its maximum working concentration (8 µM) in the present study. These results suggest the safety of 2′,4′-DHC against mammalian hosts. Accordingly, the results showed that maximum working concentration (8 µM) of 2′,4′-DHC did not affect the growth of *V. vulnificus* strain MO6-24/O, as demonstrated by the growth curve (Figure S4A). The working concentrations (maximum up to 8 µM) of 2′,4′-DHC was non-inhibitory to *V. vulnificus* growth because it is 16- fold lower than the MIC concentration against *V. vulnificus* (128 µM).

### 2.4. Assessment of Antivirulence Activity Using qRT-PCR and Phenotype

**Figure 3:**
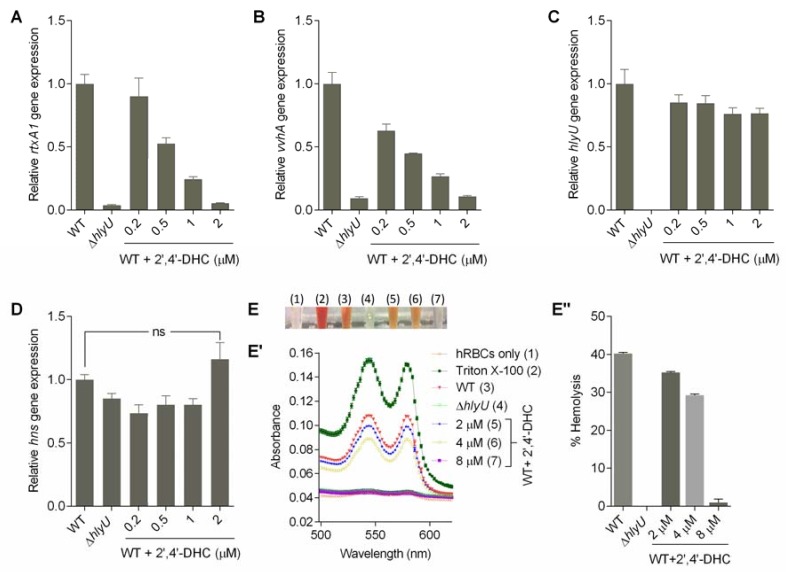
Effect of 2′,4′-DHC on the expression of HlyU-regulated genes and on the virulence phenotype of *V. vulnificus*. (**A**,**B**) Effect of 2′,4′-DHC treatment on gene expression of HlyU-regulated toxins (*rtxA1* and *vvhA*) and (**C**,**D**) global regulators (*hlyU*, *hns*) in the WT *V. vulnificus.* The expression of the target genes in the ∆*hlyU* strain was also monitored as a negative control. The target gene expression was normalized with the endogenous control gene (gyrB). The relative expression level of target gene against untreated WT sample is displayed; (**E**) inhibitory effect of 2′,4′-DHC treatment on the hemolysis activity of *V. vulnificus*. Inhibitory effect of 2′,4′-DHC on the hemolysis activity is visualized by the color of lysed hRBCs in e-tubes. The number in brackets represent the hemolysis conditions mentioned in E’; (E’) qualitative analysis of hemolysis inhibition shown by absorption spectra (500 to 650 nm) of chemical treated and untreated hRBCs; and (**E**’’) inhibitory effect of 2′,4′-DHC on the hemolysis activity is quantified by percent hemolysis that is expressed as 100 × (A_540_ of hRBCs treated with inhibitor/A_540_ of hRBCs treated with 1% Triton X-100).

The relative gene expression analysis was performed at 0.2, 0.5, 1 and 2 µM concentrations of 2′,4′-DHC (Figure 3A–D). At this concentration range, the HlyU transcript remained unchanged, indicating that 2′,4′-DHC does not interfere with the upstream regulators of HlyU under the tested conditions (Figure 3C). 2′,4′-DHC significantly reduced the transcription of major cytolytic toxins *rtxA1* (repeat-in-toxin) and *vvhA* (hemolysin) (Figure 3A,B) without a significant change in *hns* gene expression (Figure 3D). The antivirulence properties of 2′,4′-DHC were further evaluated by assessing the hemolytic activity contributed by both RtxA1 and VvhA under in vitro conditions. The hemolytic activity of *V. vulnificus* was found to be visibly decreased in a concentration-dependent manner, and 8 µM of 2′,4′-DHC reduced the hemolysis equivalent to hemolytic activity of the ∆*hlyU* strain (Figure 3E,E”). The decline in the HlyU regulated toxin gene transcripts and unchanged *hlyU* gene expression indicated that the mechanism of action of 2′,4′-DHC is presumably via inhibition of HlyU interaction with its cognate DNA.

### 2.5. Mechanism of Action of 2′,4′-DHC and Its In Vivo Validation 

**Figure 4:**
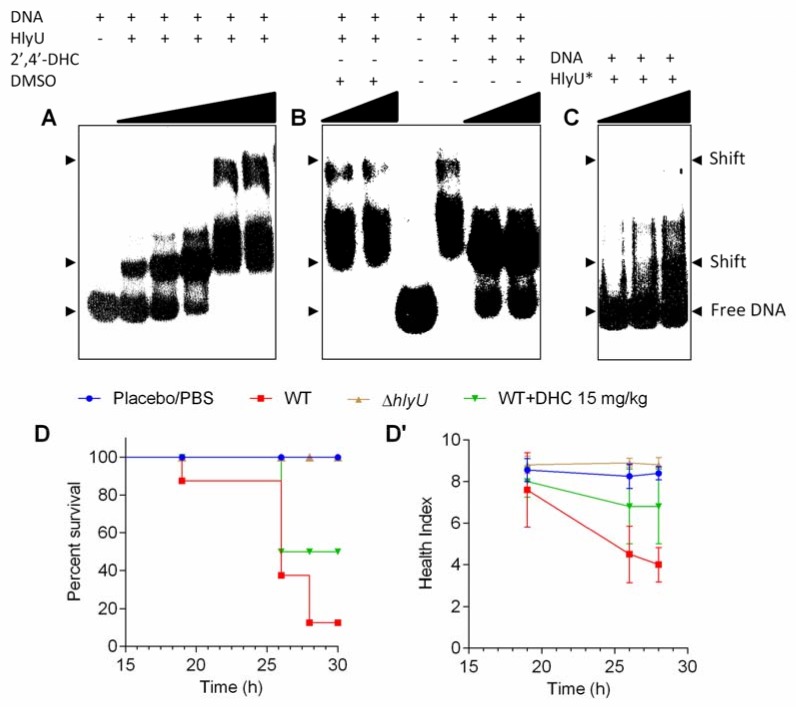
Electrophoretic mobility shift assay and in vivo validation of 2′,4′-DHC with *G. mellonella* infection model. (**A**) Titration of 3 nM (261 bp) P*_rtxA_*_1_ DNA test probe with increasing concentrations (25, 50, 100, 150 and 300 nM) of HlyU protein. (**B**) Effect of 2′,4′-DHC on HlyU binding to cognate P*_rtxA_*_1_ DNA. A total of 150 nM HlyU protein was incubated with varying concentrations of 2′,4′-DHC (150 and 300 µM) or DMSO control for 15 min and then DNA was added. (**C**) Interaction of HlyU dimerization mutant L91A/L17A protein (HlyU*) with the P*_rtxA_*_1_ DNA test probe. The concentrations of HlyU* were 40, 100 and 200 nM. The DNA protein mixtures were incubated for 20 min at room temperature (25 ± 2 °C) in 1× binding buffer, before loading onto 6% DNA retardation gel. Free DNA and shifts are indicated with arrow heads. (**D**) Kaplan–Meier survival curves of *G. mellonella* larvae treated with 15 mg/kg of 2′,4′-DHC or controls in the *V. vulnificus* infection model. (**D’**) Health index of larvae plotted as the average of every group (10 larvae/group) at each time point.

The WT HlyU protein exists as a dimer, and its dimerization mutant (HlyU*) was achieved by mutating leucine 91 and 17 residues with alanine (L91A/L17A), as reported previously [22]. Freshly prepared WT HlyU protein with P^32^-labelled P*_rtxA_*_1_ (261 bp) showed two shifts at higher concentrations of HlyU protein in Electrophoretic Mobility Shift Assay (EMSA) (Figure 4A). The higher and seemingly specific second shift disappeared after 2′,4′-DHC treatment and the free DNA probe was released (Figure 4B). This second shift did not appear with the dimerization point mutant, HlyU* protein (Figure 4C), indicating the disrupted ability of HlyU to interact with the DNA, presumably by interfering with the dimerization interface or with the DNA binding interface of HlyU. These data showed that 2′,4′-DHC can inhibit the DNA binding ability of HlyU under our experimental conditions, wherein the first shift was visible and remained unchanged in both HlyU* and inhibitor treatment. Based on the experimental evidence, the inhibition of HlyU activity will impede the de-repression of the invasive toxin network under its control. The antivirulence activity of 2′,4′-DHC was validated in *Galleria mellonella* infection model. *V. vulnificus* was injected into the haemocoel of *G. mellonella* larvae and after an hour a single dose of 2′,4′-DHC (15 mg/kg) was administered. As a result, 2′,4′-DHC averted the pathophysiology of *V. vulnificus* and showed ~50% larvae protection with a relatively better health-index based on their motility, cocoon formation, melanization, and survival, as compared to 10% larvae survival in the no treatment infection group (Figure 4D,D’).

## 3. Discussion

The fact sheet from the World Health Organization states, “Food safety, nutrition and food security are inextricably linked. Unsafe food creates a vicious cycle of disease and malnutrition, particularly affecting infants, young children, elderly and the sick” [23]. AMR *Vibrio vulnificus* contaminates nutrient-rich seafood and is extremely efficient in evading the host immune system and antibiotic treatments and thus causes a high rate of fatality. Most of the antivirulence approaches that are applied for bacterial pathogens target solitary virulence genes. As expected, such approaches show only marginal protection effects under in vivo infection conditions. To overcome this limitation, we focused on the master virulence regulator, HlyU, which is known to induce multiple virulence factors during infection. The antivirulence strategy targeting HlyU effectively disarmed the most primary and potent virulence factors (toxin network) and accessory multiple virulence factors. 2′,4′-DHC was identified using a newly developed orthogonal heterologous and integrated stable inhibitor/drug assay platform targeting HlyU (Figure 1) which possesses several advantages: (a) heterologous reporters make an isolated system to avoid the signal interference due to the cross-talk of transcription factors/regulators; (b) genomic integration of the scorable reporter provides a consistent signal compared to the two/multiple plasmid-based reporter platform; (c) avoidance of the use of multiple antibiotics to maintain plasmids in the reporter strain, which might hamper the signal due to the degradation of antibiotics and consequent loss of plasmids; and (d) ease of handling a non-pathogenic inhibitor assay platform (Figures S1 and S2, Figure 1). 

Three antivirulence natural products, namely resveratrol 4′-methyl ether, curcumin, and 2′,4′-DHC, were identified in the primary screening (Table S3). Resveratrol modulates host–microbe interaction and reduces cytotoxicity by inhibiting the bacterial motility and transcription of the *rtxA1* toxin in *V. vulnificus*. However, the mechanism of reduced transcription of *rtxA1* has not been elucidated [19]. Curcumin is also reported to inhibit the *V. vulnificus* quorum sensing and adhesion to the host cells. It also protects the host cell from damage caused by *V. vulnificu*s such as actin aggregation and NF-kB translocation [20]. However, the antivirulence mechanism of the action of curcumin is through hitherto unknown mechanisms, and the target of curcumin in *V. vulnificus* is yet to be identified. The identification of previously known antivirulence chemicals, resveratrol 4′-methyl ether and curcumin, using this orthogonal reporter platform suggests that the mechanism of action of these two chemicals is directly or indirectly related via HlyU or its upstream regulatory pathway(s). 

Among other hits, an interesting commonality was observed between isoliquiritigenin and 2′,4′-DHC hit compounds: (a) a chalcone backbone and (b) the mild growth inhibition of *V. vulnificus* at higher concentrations (Figures S3 and S4, Table S3). Nevertheless, 2′,4′-DHC practically shuts down the rtxA1 transcription and was estimated to be equivalent to the ∆*hlyU* strain (>30-fold inhibition) at a non-growth inhibitory concentration (5 µM), whereas isoliquiritigenin at 10 µM inhibited transcription of rtxA1 only up to 2.5-fold. These results clearly indicate that the mild bacteriostatic nature of chalcones at higher concentrations against *V. vulnificus* is not the reason for the reduced transcription of major toxin genes (*vvhA* and *rtxA1*); rather the antivirulence activity of 2′,4′-DHC is specific and presumably occurs via targeting the master VTF, HlyU (Figure 3). The natural product 2′,4′-DHC is a flavonoid from a plant source that consists of a basic chalcone moiety comprising two aromatic rings linked covalently by an unsaturated carbonyl short chain (Figure 2B).

The HlyU regulated cytolytic toxins are the prerequisite virulence factors in the establishment and progression of *V. vulnificus* pathogenesis [5]. The RtxA1 toxin destabilizes the cytoskeleton of the host cells leading to the death of host cells [14,18]. Moreover, hemolysin upregulates the autophagy via the lipid raft-mediated c-Src/NOX signaling pathway and ERK/eIF2α activation [24]. Additionally, recombinant VvhA has recently been shown to induce NF-*k*B-dependent mitochondrial cell death via the production of reactive oxygen species mediated by lipid rafts in intestinal cells [25]. Both RtxA1 and VvhA toxins contribute to the hemolysis of hRBC, mostly for iron acquisition to maintain pathogenicity. 2′,4′-DHC showed dominant inhibition of *rtxA1* and *vvhA* transcripts as well as haemolysis phenotype (Figure 3). Therefore, the antivirulence natural compound, 2′,4′-DHC, is expected to support the host defense by inhibiting the *V. vulnificus* toxins and self-destructing autophagy via various signaling pathways as reported earlier [24,25].

The mechanism of action of 2′,4′-DHC is elucidated via inhibition of the DNA–HlyU protein interaction. The EMSA results showed the precise targeting of the known master virulence factor, HlyU (Figure 4A–C). We established a *G. mellonella* (wax-worm) infection model for studying *V. vulnificus* pathogenesis in an earlier study [12]. In this model, 2′,4′-DHC protected ~50% wax-worm larvae from *V. vulnificus* infection (Figure 4D,D’). The *G. mellonella* infection model possesses both the humoral and innate immune systems, which is an attractive, simple, and easy to handle infection model with no biosafety or ethical issues. For prospective studies, 2′,4′-DHC can be verified in the mouse model to assess the antivirulence activity and be derivatized, if required, to improve the ADME in mammalian hosts for an effective antivirulence agent to treat *V. vulnificus* infections.

## 4. Materials and Methods 

### 4.1. Strains and Culture Conditions

*V. vulnificus* MO6-24/O [26] (hereafter termed as *V. vulnificus*) and ∆*hlyU* deletion mutant [27] strains were routinely cultured in Luria–Bertani (LB) medium supplemented with 2% sodium chloride (LBS) or on LBS solid agar plates (1.5% agar) at 37 °C. The growth of *V. vulnificus* was assessed by measuring turbidity at 600 nm (OD_600_) and counting the colony forming units (CFU). *E. coli* strains were grown at 37 °C in LB medium (broth/agar) with the appropriate concentrations of antibiotics (ampicillin, Amp at 100 µg/mL; kanamycin, Km at 100 µg/mL; and chloramphenicol, Cm at 33 µg/mL) to select for recombinant strains. All strains and plasmids used in the present study are listed in Table S1. 

### 4.2. Development of Orthogonal Reporter Platform

All recombinant DNA techniques were performed using various molecular biology kits for plasmid and genomic DNA isolation (Intron Biotech, Seongnam, Korea), and gel purification of DNA (Cosmogenetech LaboPass, Gel extraction kit, Seoul, Korea) as per the manufacturers’ protocols. An expand long PCR amplification kit (Qiagen, Hilden, Germany) was used to amplify the long DNA cassette (P*_rtxA_*_1_*::luxCDABE_cm^r^*, 7.997 kb) [12] harboring the P*_rtxA1_* promoter tagged with *luxCDABE* genes and chloramphenicol acetyltransferase gene using specific primers (Table S1). The ~8 kb long region was flanked with a λ-red recombinase site for the genomic integration as described earlier [28]. Briefly, the DpnI treated linear DNA fragment was ethanol precipitated and washed twice with 70% ethanol. The linear DNA was electroporated [29] (BIORAD Gene Pulser X-cell) into the *E. coli* MG1655 strain [30] harboring pKD46 and expressing λ-red recombinase under the arabinose inducible promoter. The *hlyU* gene was PCR amplified from the *V. vulnificus* genome and cloned at the SalI and SacI sites (Table S2) of the modified *pmalc2x_km^r^* plasmid [31] (*km^r^*, *amp^s^*) to achieve pMAL-c2X*_km^r^_hlyU.*

### 4.3. Screening of the Natural Product Library and Antivirulence Lead Compound Identification

The natural product library (800 compounds) was screened with the pMAL-c2X_*km^r^*_*hlyU* in *E. coli-*P*_rtxA_*_1_*::lux_cm^r^* (reporter strain) in 48-well plate format at 20 µM. Overnight grown cultures were diluted by 1:500 in fresh LB medium supplemented with appropriate antibiotics and 1 mM IPTG. Each plate was complemented with the *E. coli* P*_rtxA_*_1_*::lux_cm^r^* strain with an empty pMAL-c2X_km*^r^* plasmid as the negative control for background signal assessment. 

### 4.4. Quantitative Real-Time PCR (qRT-PCR) 

Overnight *V. vulnificus* cultures were adjusted to obtain an initial 2 × 10^6^ CFU/mL bacterial count. The WT strain was supplemented with or without secondary screening hit compounds or 2′,4′-DHC or DMSO in 3 mL fresh LBS medium at concentrations that were assessed as being non-growth inhibitory. The cultures were allowed to grow up to OD_600_ ~1.8–2.0. The ∆*hlyU V. vulnificus* strain was included as the control. RNA was isolated using a QIAGEN RNeasy Mini kit and cDNA was synthesized using the Ecodry Premix (random hexamer) kit (Takara Bio, Mountain View, CA, USA) as described earlier [12]. The qRT-PCR reactions were performed using Taq Universal SYBR Green supermix (Bio-Rad, Hercules, CA, USA) to determine the relative expression of *rtxA1*, *vvhA*, *hlyU*, and *hns* genes wherein the *gyrB* gene served as an endogenous control. Relative gene expression was analyzed using the 2^−ΔΔCT^ method [32].

### 4.5. Host and Pathogen Cytotoxicity by Small Molecule 2′,4′-DHC

The toxicity of lead antivirulence small molecule 2′,4′-DHC was evaluated against HeLa and HEK293 human cell lines using an EZ-Cytox cell viability assay kit (DoGen, Seoul, Korea) as described earlier [12]. HeLa and HEK293 cells were obtained from the American Type Culture Collection (ATCC) and were cultured in Dulbecco’s modified Eagle’s medium and 10% fetal bovine serum at 37 °C with 5% CO_2_. The minimum inhibitory concentration (MIC) of 2’,4’-DHC was determined according to the Clinical and Laboratory Standards Institute (CLSI, 2012) guidelines in Mueller Hinton (MH) broth [33]. 

### 4.6. Hemolysis Assay

The hemolysis assay was performed using hRBCs as described previously [34]. Briefly, overnight cultures of WT *V. vulnificus* strains were diluted in fresh LBS medium, treated with DMSO or 2′,4′-DHC, and incubated for 3 h. The culture supernatant was diluted half in phosphate-buffered saline (PBS, pH 7.4) before mixing with 1% hRBC suspension in PBS and incubated at 37 °C under shaking conditions for 2.5 h. After incubation, the reaction mixture was centrifuged and the hemolysis was determined by measuring the absorbance of the supernatant at 540 nm (A_540_). Triton X-100 (1%) treated hRBC suspension was used as a 100% hemolysis control and the percent hemolysis was calculated as described earlier [34].

### 4.7. Cloning, Expression, and Purification of WT and Dimerization Mutant (L91A/L17A) of V. vulnificus HlyU 

The 297 bp HlyU coding region was directionally cloned at BamHI and XhoI site of pProEx-HTb vector to express the recombinant HlyU with N-terminus His_6_ tag (Invitrogen). The dimerization mutant protein of HlyU (L91A/L17A) [22] was generated by site-directed mutagenesis PCR [35] (see Table S2 for primers) and confirmed by sequencing. The recombinant HlyU and L91A/L17A point mutated (HlyU*) protein was purified as described earlier [36]. Briefly, His_6_-HlyU WT and the point mutant were expressed in *E. coli* BL21 (DE3) and grown until OD_600_ 0.4–0.5 for induction with 1 mM IPTG and incubated at 16 °C for 8 h. Cells were harvested and purified by Ni-NTA and size exclusion chromatography (GE Healthcare) in a final buffer containing 25 mM Tris-Cl (pH 7.5) and 100 mM NaCl. The protein purity was verified by sodium dodecyl sulfate–polyacrylamide gel electrophoresis (SDS-PAGE).

### 4.8. Electrophoretic Mobility Shift Assay (EMSA)

For EMSA, 261 bp DNA from the *rtxA1* promoter region (P*_rtxA1_*) was amplified from the genomic DNA of *V. vulnificus* MO6 and labelled with γ-ATP^32^. DNA and protein were mixed in 20 µL by adding 3 nM radio labelled probe with varying concentrations of protein in 1× binding buffer (100 mM HEPES, pH 7.6, 5 mM EDTA, 50 mM (NH_4_)_2_SO_4_, 5 mM dithiothreitol, 1% (*v*/*v*) Tween 20, and 150 mM KCl) [17]. For the inhibitor study, 2′,4′-DHC was added to the protein 15 min prior to the addition of the labelled DNA. The DNA-protein binding reactions were performed at room temperature (25 ± 2 °C) for 20 min and loaded on pre-equilibrated 6% DNA retardation gel. The gel was electrophoresed at 150 V at room temperature, followed by drying and exposure to the film at −80 °C for development.

### 4.9. Galleria mellonella Infection Model for V. vulnificus

Larvae of *G. mellonella* (130 ± 30 mg) were purchased from a local vendor in Korea. *V. vulnificus* strains were sub-cultured for 6 h and the bacterial cells were washed and resuspended in phosphate buffer saline (PBS, pH 7.4). A total of 200–300 CFU of *V. vulnificus* was injected into the hemocoel of larvae through the left posterior proleg. The antivirulence 2′,4′-DHC (20 µL, 15 mg/kg) was administered via the right posterior proleg after 1 h of infection [37,38], and the larvae were monitored for up to 30 h for survival. Ten larvae were used per group. PBS (pH 7.4) was used as the placebo control to observe the effect of injection trauma. A ‘no manipulation’ control was maintained in each experiment. Health index was plotted as average of each group by monitoring activity, melanization, cocoon formation and survival of larvae [39].

## 5. Conclusions

The development of an elegant, isolated, and noise-free orthogonal inhibitor screening platform identified 2′,4′-DHC as a novel, high potential antivirulence natural product that protected the host from *V. vulnificus* pathogenesis by inhibiting the HlyU mediated toxin-network necessary for establishing *V. vulnificus* infection.

## Supplementary Materials

The following are available online at www.mdpi.com/1420-3049/23/6/1492/s1.
