# Supplementary material for: Identification of 2′,4′-Dihydroxychalcone as an Antivirulence Agent Targeting HlyU, a Master Virulence Regulator in Vibrio vulnificus
Source: Molecules. 2018 Jun 20;23(6):1492. doi: 10.3390/molecules23061492 (PMC6099652; doi:10.3390/molecules23061492)
Supplement: Supplementary file 1 [file molecules-23-01492-s001.pdf]

# Supporting information

**Article Title:**

**Development of an Orthogonal Inhibitor Screening Platform and Identification of 2′,4′-Dihydroxychalcone Targeting HlyU, a Master Virulence Regulator in *Vibrio vulnificus***

**Authors**

Saba Imdad, Nayab Batool, Subhra Pradhan, Akhilesh Kumar Chaurasia* and Kyeong Kyu Kim*

**Affiliation**

Department of Molecular Cell Biology, School of Medicine, Samsung Medical Center, Sungkyunkwan University, Suwon, 16419, Korea

**Keywords:** HlyU inhibitor, virulence factors, toxin-network, *Vibrio vulnificus*, 2′,4′-dihydroxychalcone, foodborne infectious disease, *Galleria mellonella* infection model, orthogonal screening reporter platform

* **Corresponding authors**

Email: chaurasia@skku.edu, kyeongkyu@skku.edu

Tel: 82-31-299-6136

Fax: 82-31-299-6159

# Contents:

1. **Supplementary Figures**

   Figure S1. Plasmid maps

   Figure S2. Multiple plasmid-based reporter system versus stable inhibition reporter platform.

   Figure S3. Screening of primary antivirulence hits for growth inhibition of *V. vulnificus*

   Figure S4. Effect of 2',4'- DHC on the growth of *V. vulnificus*

   Figure S5. *In vitro* cytotoxicity of 2',4'- DHC against cell lines

2. **Tables**

   Table S1. Strains and plasmids

   Table S2. List of primers

   Table S3. List of antivirulent and antimicrobial hits

3. **References**

## 1. Supplementary Figures

**Figure S1.**

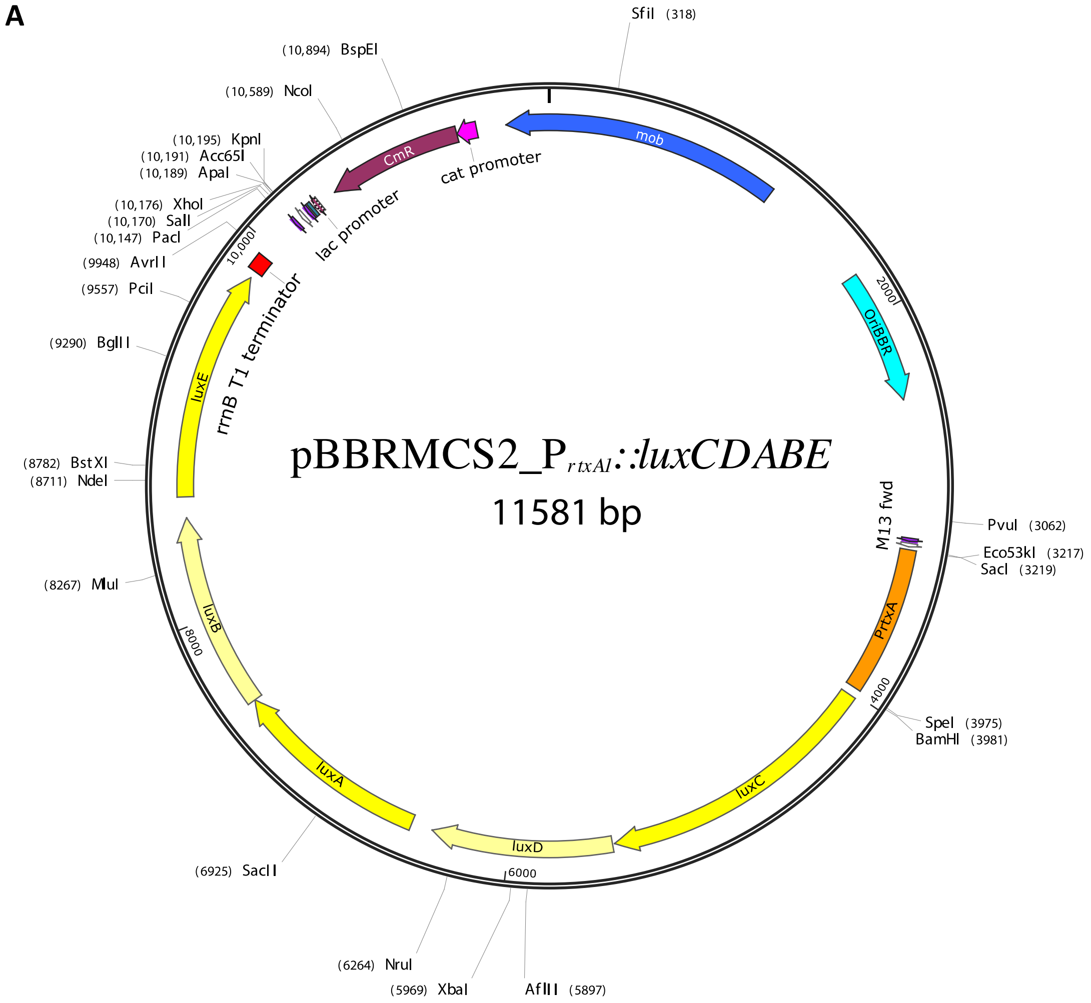
A
pBBRMCS2_P$_{rtxA1}$::luxCDABE
11581 bp
SfiI (318)
(10,894) BspEI
(10,589) NcoI
(10,195) KpnI
(10,191) Acc65I
(10,189) ApaI
(10,176) XhoI
(10,170) SalI
(10,147) PacI
(9948) AvrII
(9557) PciI
(9290) BglII
(8782) BstXI
(8711) NdeI
(8267) MluI
(6925) SacII
(6264) NruI
(5969) XbaI
AflII (5897)
SpeI (3975)
BamHI (3981)
PvuI (3062)
Eco53kI (3217)
SacI (3219)
mob
cat promoter
CmR
lac promoter
rrnB T1 terminator
OriBBR
M13 fwd
PrtxA
luxC
luxD
luxA
luxB
luxE
2000
4000
6000
8000
10,000

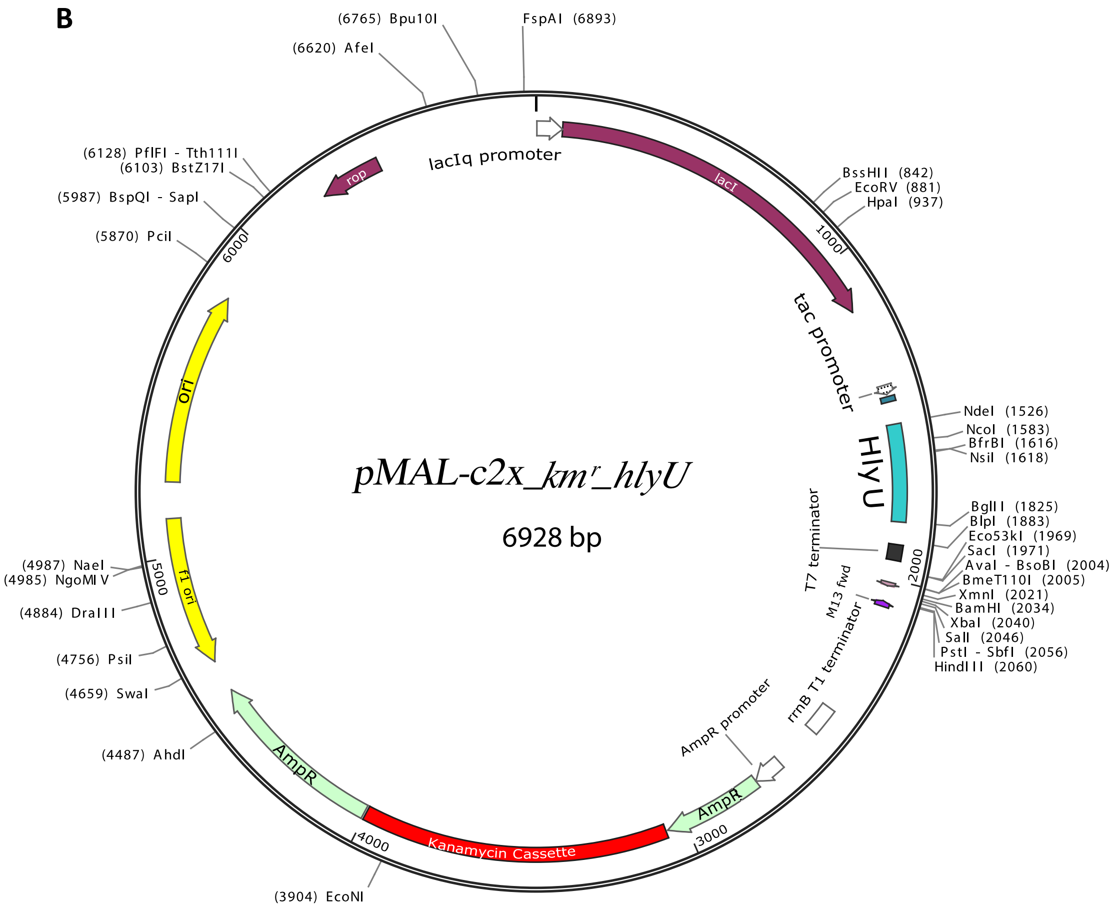


**Figure S1.** Plasmid maps. (**A**) The plasmid pBBRMCS2_$P_{rtxA1}$::*luxCDABE* map was achieved by inserting the $P_{rtxA1}$ promoter in the pBBR1MCS2-*lux* plasmid backbone [1]. The scorable reporter $P_{rtxA1}$-*luxCDABE*-$cm^r$ cassette (~8 kb; reporter fragment) was PCR amplified from pBBRMCS2_$P_{rtxA1}$::*luxCDABE* plasmid and integrated at an innocuous site of *E. coli* K-12 strain MG1655 genome and the strain was designated as *E. coli*-$P_{rtxA1}$::*lux_*$cm^r$. (**B**) HlyU virulence transcription factor encoding gene *hlyU* was cloned at SalI and SacI into pMAL-c2X expression vector [2] to obtain pMAL-c2*X_hlyU* plasmid ($amp^r$). The plasmid was modified and the kanamycin resistance marker was inserted at the ScaI site of ampicillin to inactivate the ampicillin resistance gene ($km^r$, $amp^s$). The pMAL-c2X_*hlyU_*$km^r$ underwent electroporation in the *E. coli*-$P_{rtxA1}$::*lux_*$cm^r$ strain to develop the stable inhibitor screening platform.

**Figure S2.**

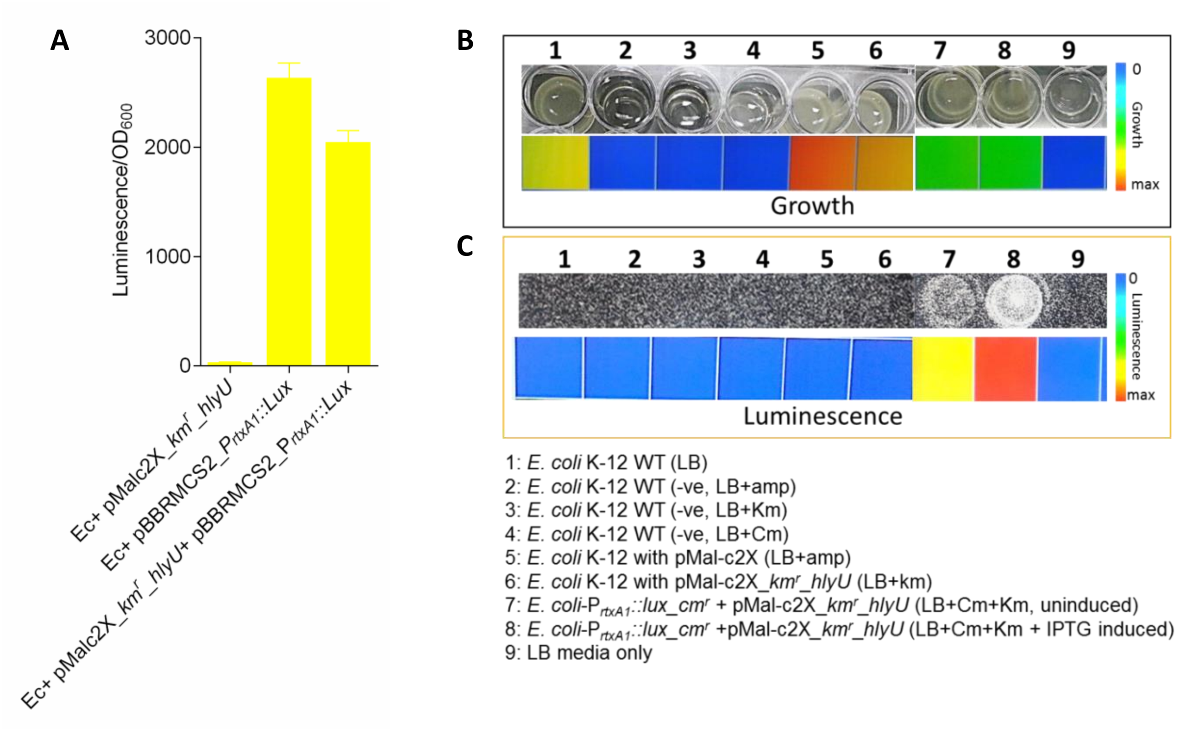


**Figure S2.** Multiple plasmid-based reporter system versus stable inhibitor reporter platform. Overnight grown culture was diluted in fresh LB medium at 1:500 ratio and supplemented with 1 mM IPTG and appropriate antibiotics (Cm and Km). The luminescence per unit OD600 was calculated after 5 h of incubation. (**A**) Quantitative expression of dual-plasmid inhibitor screening reporter system. Strain *E. coli* K-12 MG1655 is designated as 'Ec'. Two plasmids- based reporter platform became unstable and showed low luminescence either by plasmid instability or reduced antibiotic pressure to maintain the vertical transfer during assay. (**B, C**) An orthogonal stable inhibitor reporter platform. Verification of growth (**B**) and luminescence (**C**) of WT, empty/*hlyU* vector control and inhibitor screening reporter *E. coli* strains in various culture conditions are indicated as 1-9. Strain 7 showed luminescence signal due to the leaky expression of HlyU, which is enhanced upon IPTG induction (strain 8). The signal was expected to reduce when an inhibitor interacts and inhibit the DNA-binding activity of HlyU virulence transcriptional regulator.

**Figure S3.**

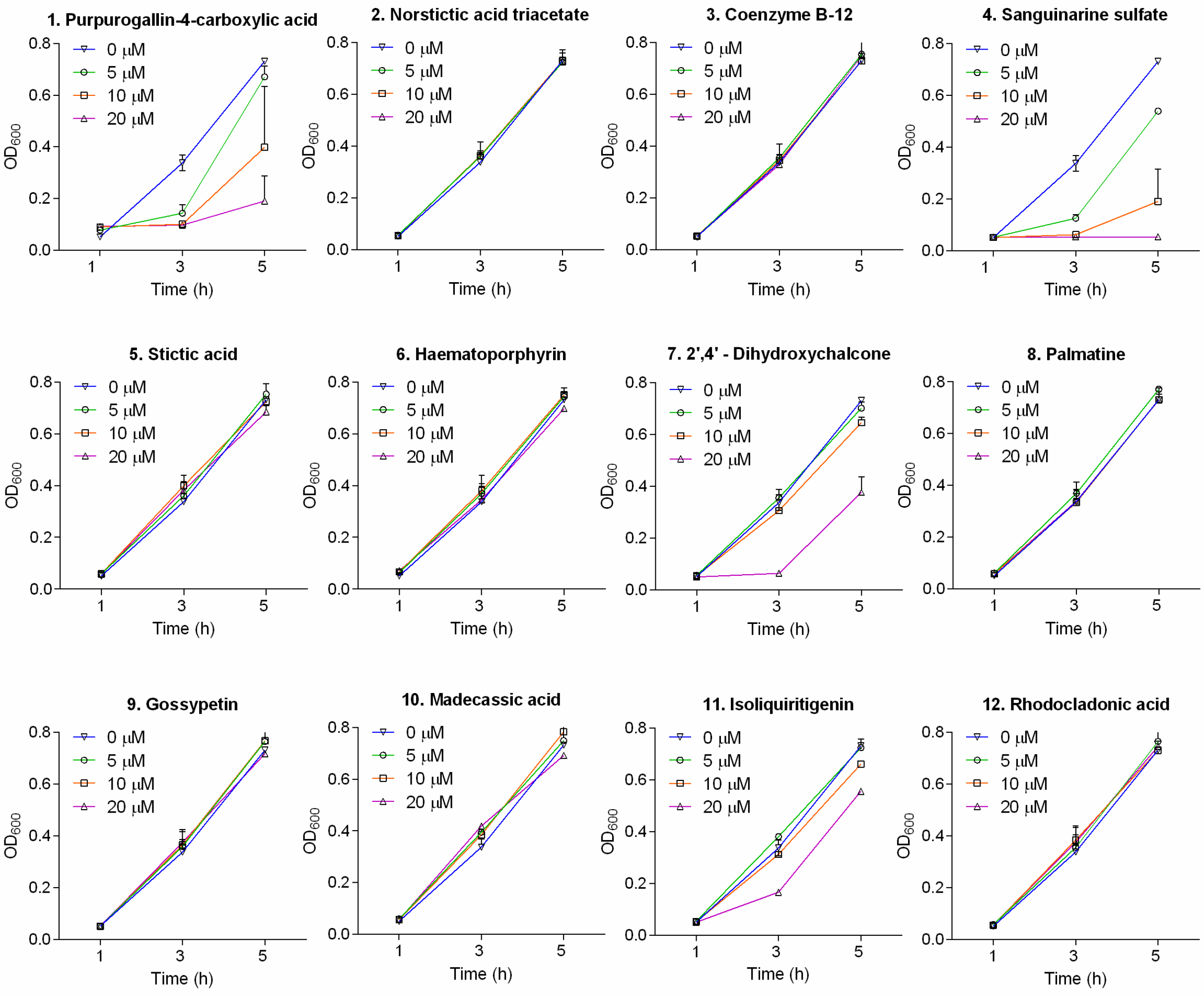


**Figure S3.** Screening of primary antivirulence hits for growth inhibition of *V. vulnificus*. The 12 antivirulence hits obtained from the *E. coli*-$P_{rtxA1}$::*lux_cm*$^{r}$+pMal-c2X_*km*$^{r}$*_hlyU* reporter were examined for dose-dependent growth inhibitory effects (secondary screening) on the target organism, *V. vulnificus*. Overnight grown culture of WT *V. vulnificus* was diluted by 1:100 in fresh LBS and incubated with 0, 5, 10, and 20 μM of antivirulence hits and $OD_{600}$ was measured in a time dependent manner. (1) Purpurogallin-4-carboxylic acid and (4) Sanguinarine sulfate were excluded from further study because of growth inhibitory effects. The remaining 10 antivirulence hits were considered secondary hits and evaluated further using qRT-PCR.

**Figure S4.**

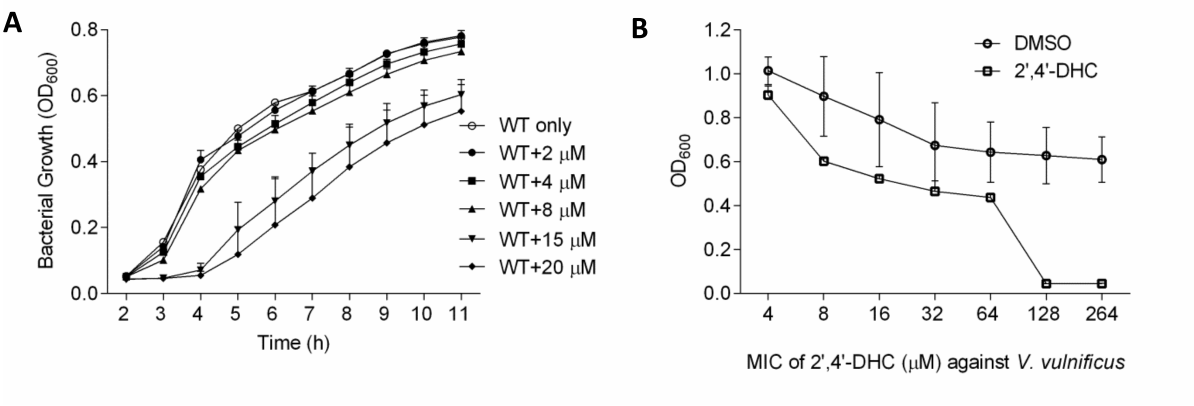


**Figure S4.** Effect of 2′,4′- DHC on the growth of *V. vulnificus.* (**A**) Growth pattern of *V. vulnificus* with different concentrations of 2′,4′- DHC. Freshly grown culture of WT *V. vulnificus* was supplemented with 2, 4, 8, 15, and 20 µM of 2′,4′- DHC and the growth response was recorded in 48-well plates by measuring $OD_{600}$ over time in LBS medium. (B) Minimum inhibitory concentration (MIC) of 2′,4′- DHC against *V. vulnificus.* MIC was determined with an initial inoculum of $8 \times 10^5$ CFU/mL in MH broth and the observation was recorded after 18 h of incubation according to the CLSI protocol [3].

**Figure S5.**

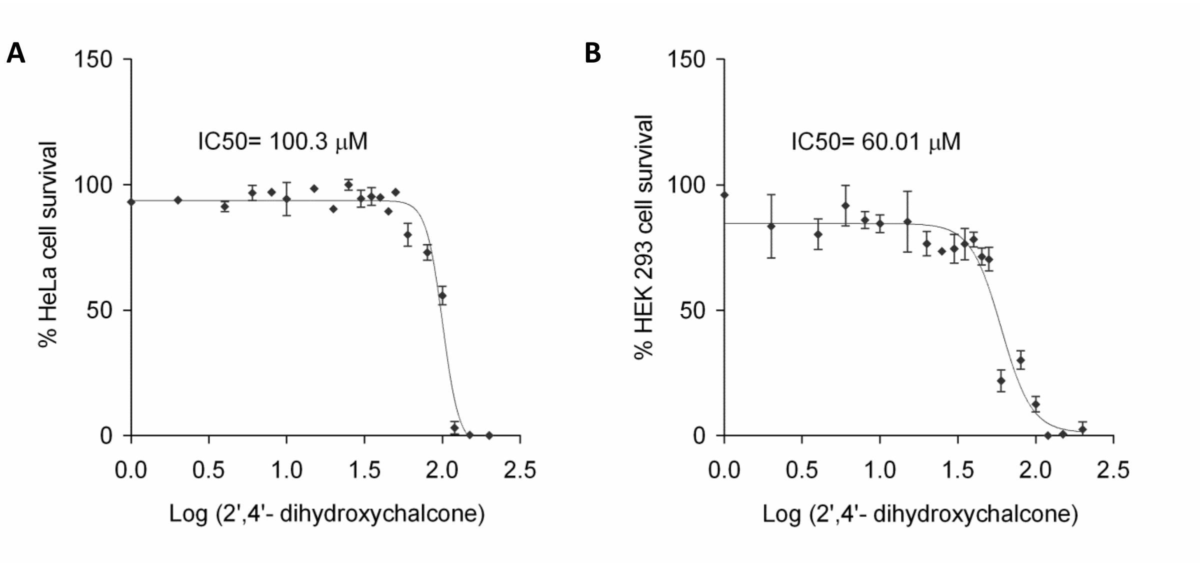


**Figure S5.** *In vitro* cytotoxicity of 2′,4′- DHC against cell lines. Toxicity of 2′,4′- DHC was tested with a EZ-Cytox kit for (**A**) HeLa cells and (**B**) HEK293 cells. The cytotoxicity was measured as a function of cellular viability. A total of 2 × $10^4$ HeLa cells (in DMEM and 10 % FBS medium) were incubated with varying concentrations of 2′,4′- DHC (1–200 µM) for 48 h at 37°C with 5% $CO_2$. Absorbance ($A_{450}$) was measured using a multi-plate reader (Tecan Infinite M200), after adding the kit solution. The cell culture media was used as blank and the cells with DMSO treatment were considered as 100% viable. GraphPad Prism was used to determine the IC50 [1].

## 3. Tables

**Table S1. Strains and plasmids**

| Plasmid/Strain | Description | Reference |
|---|---|---|
| **Plasmids** | | |
| pMAL-c2X | Cytoplasmic expression plasmid for cytoplasm, *amp*$^r$ | [2] |
| pMAL-c2X_*hlyU* | HlyU cloned at SalI and SacI site in pMAL-c2X plasmid (*amp*$^r$) | This study |
| pMAL-c2X_*km*$^r$_*hlyU* | pMAL-c2X_*hlyU* resistance marker changed to kanamycin (*km*$^r$ , *amp*$^s$) | This study |
| pProEx-HTb | N-termial $His_6$ protein overexpression (*amp*$^r$) | Lab stock |
| pProEx-HTb_*hlyU* | *hlyU* cloned at BamHI (F) and XhoI (R) site in pProEx-HTb (*amp*$^r$) | This study |
| pProEx-HTb_*hlyU** | L91A/L17A point mutated *hlyU** (*amp*$^r$) | This study |
| pBBRMCS2_$P_{rtxA1}$::*luxCDABE* | *E. coli* with pBBRMCS2_$P_{rtxA1}$::*luxCDABE* (*cm*$^r$) | [1] |
| pKD46 | λ-red recombinase encoding plasmid under arabinose inducible promoter, temperature sensitive origin of replication (*amp*$^r$) | [4] |
| **Strains** | | |
| *E. coli* DH5α | $F^-$ Φ80*lac*ZΔM15 Δ(*lac*ZYA-*arg*F) U169 *rec*A1 *end*A1 *hsd*R17 ($rK^-$, $mK^+$) *phoA supE44 λ– thi-1 gyrA96 relA1* | Lab stock |
| *E. coli* BL21(DE3) | *E. coli str*. B F– ompT gal dcm lon hsdSB(rB–mB–) λ(DE3 [lacI lacUV5-T7p07 ind1 sam7 nin5]) [malB+]K-12(λS) | Lab stock<br>Lab stock |
| *E. coli* K-12 MG1655 | Wild-type (WT) *E. coli* strain | [5] |
| *E. coli*-$P_{rtxA1}$::*lux_cm*$^r$ | Reporter cassette; chromosomal integration of ~8 kb $P_{rtxA1}$::*luxCDABE_cm*$^r$ | This study |
| *E. coli*-$P_{rtxA1}$::*lux_cm*$^r$ reporter | *E. coli*-$P_{rtxA1}$::*lux_cm*$^r$ with pMAL-c2X_*km*$^r$_*hlyU* | This study |
| WT *Vibrio vulnificus* | Wild type MO6-24/O clinical isolate | [6] |
| Δ*hlyU* | *V. vulnificus hlyU* gene deletion mutant | [7] |

## Table S2. List of primers

| Purpose | Name | Sequence |
|---|---|---|
| **Chromosomal Integration** | *Int_F* | CGCGGGGAACTCTCGGTTCAGGCGTTGCAAACCTGGCTACTGAAGTTCCTATACTTTCTAGAGAATAGGAACTTCGAGCTCGAATCAAATAAAATG |
| | *Int_R* | TAAACCGTTTGGATCGGGTCTGGAATTTCTGAGCGGTCGCGAAGTTCCTATTCTCTAGAAAGTATAGGAACTTCATGATCGGCACGTAAGAGGTTC |
| | *km*$^r$_F | ACCATAAGTACTCAGCATCGCAGTGGGAACGATGCCC |
| | *km*$^r$_R | ACCATAAGTACTTGGCCGGGGGACTGTTGGGCGCCATC |
| **Confirmation of Integration** | $P_{rtxA1}$Lux_*cm*$^r$_Intconf_F | TGGGTTTGAAAATGGGAGCTGGGAGTTC |
| | $P_{rtxA1}$Lux_*cm*$^r$_Intconf_R | TTAGCTGATCTTTAATAATAAGGAAATG |
| **HlyU cloning in pMAL-c2X-*km*$^r$** | *hlyU*-F | ACGGCGGTCGACATGAACTTAAAAGATATGGAG (SalI) |
| | *hlyU*-R | ACGGCGGAGCTCTTATTCTTCGCAATAAAGACTG (SacI) |
| **qRT-PCR** | *rtxA1*-F | GATGGTTACAAAGCCGATAC |
| | *rtxA1*-R | TCTGGGTTATCAAGCAGAAT |
| | *vvhA*-F | AGACTATCGCATCAACAACC |
| | *vvhA*-R | AAACGTCATAGTTCGGTTTG |
| | *hlyU*-F | TTCTGCTAAAGCTGTCGTATT |
| | *hlyU*-R | AAACCGTTTGTGCTTCTTTA |
| | *hns*-F | GAACAAATTGCTAAAGATGGT |
| | *hns*-R | GATTTACCCGCATCTAATTG |
| | *gyrB*-F | TCAGTTTCTGTTAGCGATGA |
| | *gyrB*-R | ATCGTCAACAGCACTTTTTC |
| **Site Directed Mutation of HlyU** | *hlyU*L91A-F | TAAAAGCAATGATTAAACTGGCTCACAGTCTTTATTGCGAAGAA |
| | *hlyU*L91A-R | TTCTTCGCAATAAAGACTGTGAGCCAGTTTAATCATTGCTTTTA |
| | *hlyU*L17A-F | CTGCTAAAGCTGTCGTATTAGCTAAAGCCATGGCCAATGAAAG |
| | *hlyU*L17A-R | CTTTCATTGGCCATGGCTTTAGCTAATACGACAGCTTTAGCAG |
| **EMSA** | $P_{rtxA1}$ probe-F | TCAAATAAAATGGCGGGTG |
| | $P_{rtxA1}$probe-R | TCAAAAACGCTGCAATAAAC |
| **HlyU-His$_6$ protein expression** | His-HlyU-F | ACGGCGGGATCCAACTTAAAAGATATGGAG (BamHI) |
| | His-HlyU-R | ACGGCGCTCGAGCTATTCTTCGCAATAAAG (XhoI) |

Restiction endonuclease shown in bracket.

**Table S3. List of antivirulent and antimicrobial hits**

| Sr. No. | Antivirulent Hits | | Antimicrobial Hits | |
|---|---|---|---|---|
| | **Compounds** | **Structure** | **Compounds** | **Structure** |
| **1.** | Purpurogallin-4-carboxylic acid | 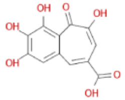 | Mupirocin | 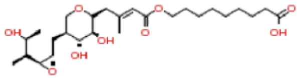 |
| **2.** | Norstictic acid triacetate | 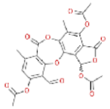 | Colistin sulfate | 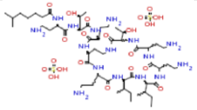 |
| **3.** | Coenzyme B12 | 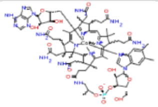 | Bleomycin | 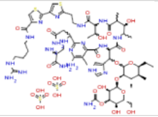 |
| **4.** | Sanguinarine sulfate | 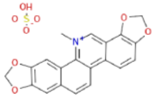 | Polymyxin B sulfate | 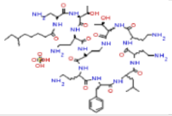 |
| **5.** | Resveratrol 4’-methyl ether | 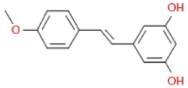 | Tetracycline hyrochloride | 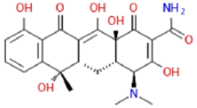 |
| **6.** | Curcumin | 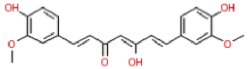 | Patulin | 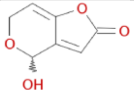 |
| **7.** | 2’,4’-dihydroxychalcone | 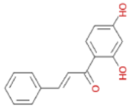 | Plumbagin | 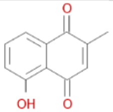 |

| | | | | |
|---|---|---|---|---|
| **8.** | Palmatine | 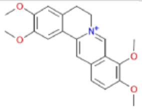 | | |
| **9.** | Gossypetin | 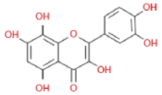 | | |
| **10.** | Madecassic acid | 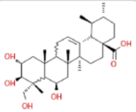 | | |
| **11.** | Isoliquiritigenin | 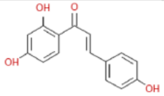 | | |
| **12.** | Rhodocladonic acid | 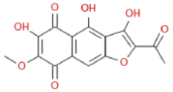 | | |
| **13.** | Stictic acid | 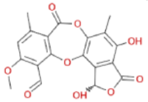 | | |
| **14.** | Haematoporphyrin | 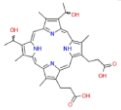 | | |

## 4. References


1. Imdad, S.; Chaurasia, A.K.; Kim, K.K. Identification and validation of an antivirulence agent targeting HlyU-regulated virulence in *Vibrio vulnificus*. *Front Cell Infect Microbiol* **2018**, *8*, doi: 10.3389/fcimb.2018.00152.
2. Walker, I.H.; Hsieh, P.C.; Riggs, P.D. Mutations in maltose-binding protein that alter affinity and solubility properties. *Appl Microbiol Biotechnol* **2010**, *88*, 187-197.
3. Wiegand, I.; Hilpert, K.; Hancock, R.E. Agar and broth dilution methods to determine the minimal inhibitory concentration (MIC) of antimicrobial substances. *Nat Protoc* **2008**, *3*, 163-175.
4. Datsenko, K.A.; Wanner, B.L. One-step inactivation of chromosomal genes in *Escherichia coli* K-12 using PCR products. *Proc Natl Acad Sci U S A* **2000**, *97*, 6640-6645.
5. Blattner, F.R.; Plunkett, G.; Bloch, C.A.; Perna, N.T.; Burland, V.; Riley, M.; Collado-Vides, J.; Glasner, J.D.; Rode, C.K.; Mayhew, G.F., *et al.* The complete genome sequence of *Escherichia coli* K-12. *Science* **1997**, *277*, 1453-1462.
6. Wright, A.C.; Simpson, L.M.; Oliver, J.D.; Morris, J.G., Jr. Phenotypic evaluation of acapsular transposon mutants of *Vibrio vulnificus*. *Infect Immun* **1990**, *58*, 1769-1773.
7. Jang, K.K.; Lee, Z.W.; Kim, B.; Jung, Y.H.; Han, H.J.; Kim, M.H.; Kim, B.S.; Choi, S.H. Identification and characterization of *Vibrio vulnificus PlpA* encoding a phospholipase A2 essential for pathogenesis. *J Biol Chem* **2017**, *292*, 17129-17143.
